# Long-Term Outcomes of Intermittent Exotropia: A Real-World Longitudinal Cohort Study of 415 Patients

**DOI:** 10.3390/medicina62030481

**Published:** 2026-03-04

**Authors:** Fatma Gul Yilmaz Cinar, Umay Guvenc, Rabia Akmaz, Yasemin Topalak, Ayse Burcu

**Affiliations:** 1 Department of Pediatric Ophthalmology and Strabismus, Ankara Training and Research Hospital, University of Health Sciences, 06340 Ankara, Türkiye; 2 Department of Ophthalmology, Ankara Training and Research Hospital, University of Health Sciences, 06340 Ankara, Türkiye

**Keywords:** intermittent exotropia, over-minus therapy, strabismus surgery, Kaplan–Meier survival, fusional control (ECS), stereoacuity, refractive change

## Abstract

*Background and Objectives*: Intermittent exotropia (IXT) is a common childhood strabismus with variable natural history and no universally accepted first-line management. We aimed to characterize long-term real-world outcomes and identify factors associated with alignment stability across surgical and non-surgical strategies. *Materials and Methods*: This retrospective single-center cohort study reviewed consecutive patients with IXT managed between January 2008 and March 2024. Clinical data included IXT subtype, prism and alternate cover test measurements, binocular control (ECS), stereopsis, refractive error, and treatments (observation, over-minus lenses, and surgery). Postoperative success was defined as ≤10 prism diopters (PD) of eso/exophoria without diplopia. Longitudinal refractive change was analyzed using linear mixed-effects modeling, and time to alignment failure (≤±10 PD at near and distance) was evaluated using Kaplan–Meier and Cox regression analyses. *Results*: A total of 415 patients were included (mean follow-up 53.2 ± 47.8 months). Over-minus therapy was used in 252 (60.7%) patients for a median of 24 months, and 61 (14.7%) achieved spontaneous alignment. At the final visit, combined near-and-distance alignment success was 41.0% (170/415). Among surgically treated patients (n = 167), motor success was 65.3% (109/167), with reoperation required in 12.6% (21/167). Kaplan–Meier analysis showed cumulative alignment survival of 0.899 at 1 year, 0.563 at 5 years, and 0.302 at 10 years (median 70 ± 4.7 months). In Cox modeling, surgery was strongly protective (HR 0.174), while older age (HR 1.040 per year) and poor baseline distance control (HR 1.421) increased the risk of failure; over-minus therapy was not independently associated with survival. Both treatment groups showed a similar myopic shift over time (β = −0.25 D/year), with no between-group difference. *Conclusions*: In this longitudinal cohort, intermittent exotropia showed a variable course, with many patients ultimately requiring surgery despite initial non-surgical management. Long-term success was more closely tied to preoperative control quality than age. These findings support an individualized, control-based approach to treatment planning and timing of intervention.

## 1. Introduction

Intermittent exotropia (IXT) is the most common form of childhood exotropia, characterized by alternating periods of ocular alignment and outward deviation, with epidemiologic studies reporting a prevalence ranging from 0.1% to approximately 3.7% in general pediatric populations [1,2]. It typically presents between the ages of 1 and 5 and is often more pronounced during distance fixation or in states of fatigue, illness, or inattention [1]. Over time, the frequency and severity of the deviation may increase, and the condition can progress to constant exotropia [1]. The pathophysiology of IXT remains incompletely understood, but impaired fusional mechanisms are thought to play a key role. Patients may maintain normal retinal correspondence and functional binocular vision during orthotropic phases, yet loss of motor fusion can lead to deterioration in stereopsis and development of persistent exotropia [3].

Currently, there is no universally accepted first-line treatment for IXT. Non-surgical approaches—including over-minus lens (OML) therapy, part-time occlusion, and orthoptic exercises—are often employed in the early stages. However, their long-term efficacy and influence on surgical outcomes remain controversial [4]. Consequently, surgery is frequently recommended—especially in cases with poor control or cosmetic concerns. While primary surgical success rates range between 70% and 80%, recurrence rates can approach 50% within three years postoperatively [2,5,6].

When surgery is indicated, bilateral lateral rectus recession (BLR) and unilateral recession–resection (R&R) are the most frequently used procedures. Long-term comparative studies have shown similar outcomes for both approaches, although specific subgroups may benefit more from one than the other [7]. The timing of surgery remains controversial: while early intervention may offer some advantages in particular populations, meta-analyses have not consistently demonstrated a benefit over later surgery [8].

In this study, we present one of the most extensive longitudinal cohort analyses of IXT to date, based on real-world clinical data. By examining the natural course, control patterns, refractive changes, and treatment outcomes over an extended follow-up period, we aim to provide a comprehensive characterization of IXT. By applying survival analysis and longitudinal modeling, we identified key risk factors for surgical failure and examined the clinical utility of over-minus therapy as a bridge strategy. This study offers valuable insights into the long-term management of IXT and contributes to individualized treatment planning.

## 2. Materials and Methods

### 2.1. Study Design and Setting

This was a retrospective, single-center cohort study conducted at the Pediatric Ophthalmology and Strabismus Unit of the University of Health Sciences, Ankara Training and Research Hospital. We reviewed the medical records of consecutive patients diagnosed with IXT and managed between January 2008 and March 2024. The study protocol adhered to the principles of the Declaration of Helsinki and received approval from the Scientific Research Ethics Committee of the University of Health Sciences Ankara Training and Research Hospital. (approval number: E25-451; date of 27 March 2025).

### 2.2. Participants

Patients were included if they met all of the following criteria:

1. A confirmed clinical diagnosis of IXT.
2. Documented age at presentation.
3. Complete ocular alignment measurements at both distance (6 m) and near (33 cm) using the prism and alternate cover test.
4. Availability of detailed preoperative, surgical, and postoperative follow-up records.
5. Minimum follow-up duration of 6 months.

Exclusion criteria were as follows:

- Restrictive or paralytic strabismus.
- Structural ocular pathologies (e.g., cataract, retinal disease).
- Prior extraocular muscle surgery before initial evaluation.
- Significant systemic or neurological comorbidities.
- Inability to attend regular follow-up visits.

### 2.3. Clinical Assessments

All ophthalmologic evaluations were primarily conducted by a team of pediatric ophthalmologists under standardized protocols. Ophthalmologic evaluation included:

1. Best Corrected Visual Acuity testing using age-appropriate methods.
2. Binocular Single Vision assessment via the Bagolini, Titmus or Randot tests.
3. Ocular motility assessment and alignment measurement using the prism and alternate cover test at distance and near fixation.
4. Occlusion testing: Parents were informed that at a subsequent visit the non-dominant eye would be occluded for 1 h to reveal the largest angle of deviation.

Control of exodeviation was graded separately at distance and near using a five-point office-based control scale (ECS-1 to ECS-5, lower scores indicating better control), aligned with the Mayo Clinic control grading system [9]. Levels 3–5 on the scale rate the duration of any spontaneous tropia during a 30 s observation period (with 5 indicating constant tropia; 3, tropia for <50% of observed time); if no spontaneous tropia is observed, the speed of recovery following dissociation is rated 0–2 (with 2 indicating recovery >5 s; 0, <1 s).

5. Dynamic retinoscopy to assess binocular accommodative function.
6. Cycloplegic refraction performed using cyclopentolate 1%.
7. Anterior and posterior segment evaluation via slit-lamp biomicroscopy and indirect ophthalmoscopy.

### 2.4. Treatments

Refractive correction was performed for all patients at baseline. Full optical correction was prescribed based on cycloplegic refraction to eliminate the contribution of uncorrected refractive error to ocular misalignment.

Observation was reserved for patients with favorable clinical profiles—defined as having reasonable fusional control (ECS scores 1–2) and preserved binocular single vision. These patients were closely monitored without additional intervention.

Over-minus therapy was considered for patients with moderate control impairment (ECS scores 2–3) who exhibited manifest deviation for less than 50% of the time during daily activities. OML were prescribed as a spherical minus addition to the cycloplegic refraction and adjusted in a stepwise manner based on each patient’s accommodative function. At each follow-up visit, binocular accommodative ability was re-evaluated using dynamic retinoscopy. The prescribed minus power was titrated to the maximum tolerated lens strength that did not disrupt accommodation, ensuring a balance between therapeutic effect and visual comfort. Patients receiving over-minus therapy were followed for at least 6 months from treatment initiation, with regular monitoring of ocular alignment, fusional control, and accommodative status.

Surgery was recommended if any of the following were present: a progressively increasing angle or frequency of deviation (ECS-3 or worse), loss of binocular vision, or cosmetic problems. The final decision was made by the patient/parents and surgeon. All of the patients were operated on under general anesthesia using the same surgical table. All surgeries were performed either directly by pediatric ophthalmologists with subspecialty training in strabismus or under their close supervision, in accordance with standardized clinical protocols implemented in our Pediatric Ophthalmology and Strabismus Unit. We performed strabismus surgery at the dosage recommended by Wright and Spiegel [10]. Surgical management consisted one of the following procedures: (1) bilateral lateral rectus recession (BLR), (2) unilateral lateral rectus recession and resection (RR), or (3) alternative procedures such as single or three-muscle with/without obliques. Surgery was based on the largest angle of deviation measured at both distance and near fixation. Reoperation was defined as any subsequent strabismus surgery performed after the index procedure.

Augmented surgery was defined as an intentional increase in surgical dosage beyond the standard amounts recommended by Wright and Spiegel. Specifically, the amount of recession and/or resection was increased by 1 mm compared with standard tables in selected patients with poor fusional control, significant preoperative deviation, or a high risk of postoperative recurrence, at the discretion of the operating surgeon. The decision to apply augmented dosing was based on the largest measured angle of deviation at distance or near fixation, and all procedures were performed under general anesthesia using a uniform surgical protocol.

Postoperative surgical success was defined as orthotropia, with residual alignment within 0–10 prism diopters (PD) of esophoria or exophoria, in the absence of diplopia.

### 2.5. Statistical Analysis

All statistical analyses were performed using IBM SPSS Statistics for Windows, Version 21.0 (IBM Corp., Armonk, NY, USA). The distribution of continuous variables was assessed for normality using both visual methods (histograms, probability plots) and analytical tests, including the Kolmogorov–Smirnov and Shapiro–Wilk tests. Additionally, skewness and kurtosis values within ±1.5 were considered indicative of normal distribution. Descriptive statistics were reported as mean ± standard deviation (SD) for normally distributed variables and as median with interquartile range (IQR) for non-normally distributed variables. Categorical variables were presented as frequencies and percentages.

Group comparisons were conducted as follows:

- For categorical variables, the Pearson Chi-square test, Fisher’s Exact test, or the Fisher–Freeman–Halton Exact test (in cases of small, expected cell counts) was used. Where appropriate, post hoc analyses included pairwise comparisons or adjusted residuals.
- For non-normally distributed continuous variables, comparisons between two independent groups were performed using the Mann–Whitney U test, while comparisons among more than two groups were assessed using the Kruskal–Wallis test. In cases of statistical significance, pairwise Mann–Whitney U tests were applied for post hoc evaluation.
- Within-subject comparisons of non-normally distributed variables over time were analyzed using the Friedman test, with Wilcoxon signed-rank tests for post hoc pairwise analyses.
- For repeated categorical measures, the Cochran’s Q test was used, followed by McNemar’s test for pairwise comparisons when appropriate.

Where multiple comparisons were conducted, Bonferroni correction was applied to adjust the significance threshold and control for type I error. A two-tailed *p*-value < 0.05 was considered statistically significant throughout.

### 2.6. Longitudinal and Survival Analyses

To evaluate longitudinal changes in refractive error, a Linear Mixed-Effects Model (LMM) was employed. This method accommodates repeated measures data and allows for missing values under the missing-at-random (MAR) assumption while accounting for both fixed and random effects. Survival analysis was conducted to estimate the time to surgical failure using the Kaplan–Meier method. Group comparisons of survival curves were performed using the Log-Rank test. To evaluate the proportional hazards assumption, log-minus-log plots were visually inspected, and Schoenfeld residuals were analyzed. Variables found to be significant in univariate survival analyses were included in a Cox proportional hazards regression model, using the Enter method. For covariates violating the proportional hazards assumption, stratified Cox models were applied. Model fit and adequacy were further evaluated using Martingale residuals and goodness-of-fit diagnostics. A Type I error threshold of 0.05 was used in all survival analyses. The statistical power of the study was calculated using G*Power software (version 3.1.9.7). The analysis demonstrated that, with an effect size of Cramer’s V = 0.176, a significance level of 5%, and a sample size of 415, the statistical power for a chi-square test with 6 degrees of freedom was 0.77. Therefore, the sample size of 415 patients in our study was considered sufficient to achieve adequate statistical power.

## 3. Results

### 3.1. Patient Demographics and Clinical Characteristics

A total of 415 patients with IXT were included. The mean age at onset was 3.7 ± 2.9 years (median 3; IQR, 3.5), and the mean age at presentation was 6.8 ± 4.9 years (median 6; IQR, 5). The mean follow-up duration was 53.2 ± 47.8 months (median 40; IQR, 63). Females comprised 58.6% of the cohort. Most patients were born at term (76.4%) via vaginal delivery (51.3%). A family history of strabismus or amblyopia was reported in 17.8% of cases. The most frequent IXT subtype was elemental (48.4%), followed by pseudo-divergence excess (33.5%), divergence excess (14.2%), and convergence insufficiency (3.9%) (Table 1).

When the age groups were subdivided into four categories, the differences in the distribution of exotropia types across age groups remained statistically significant at the 5% level. Post hoc analysis using adjusted standardized residuals revealed that convergence insufficiency (CI) was significantly more prevalent in the age groups ≤ 4 years, 7–14 years, and ≥15 years. Residual values greater than ±1.96 (95% confidence level) were considered to indicate statistically significant deviations from expected frequencies within each cell. The data demonstrated a clear age-related increase in the frequency of CI (0.7%, 6.3%, and 14.3%, respectively). CI was nearly absent in younger children, consistent with clinical observations that this subtype becomes more apparent with increasing age. Furthermore, the prevalence of the pseudo-divergence excess type was significantly higher in patients aged ≥15 years (Table 2).

### 3.2. Non-Surgical Management

Of the 415 patients with IXT, 252 (60.7%) received over-minus optical therapy, and 61 (14.7%) achieved spontaneous alignment within ±10 PD at both near and distance without any active intervention. Across the entire cohort, near-alignment success was observed in 292 patients (70.4%), distance success in 188 (45.3%), and combined near-and-distance success in 170 patients (41.0%) at the final examination.

### 3.3. Over-Minus Therapy

Of the total cohort, 252 patients (60.7%) received over-minus optical therapy (−0.75 to −2.50 D) for a median of 24 months (IQR, 12–36). The mean baseline age in this group was 11.2 ± 6.4 years. Pre-treatment deviations averaged 17.8 ± 10.3 PD at near and 22.5 ± 11.7 PD at distance.

Full alignment (≤±10 PD at both distances) was achieved in 38.1% of these patients without surgery. The likelihood of receiving OML therapy varied by IXT subtype (*p* = 0.002), with the highest rate in pseudo-divergence excess (71.9%) and the lowest in basic type (51.7%).

Among surgical cases (n = 167), 49.7% had previously used OML correction. These patients underwent surgery at a younger age (7.9 ± 3.5 vs. 11.2 ± 7.1 years, *p* = 0.001) but after a more extended observation period (1.83 ± 2.40 vs. 1.34 ± 2.07 years, *p* = 0.038), suggesting that early presentation led to prolonged conservative management before surgery. (Table 3).

### 3.4. Longitudinal Refractive Change

**Figure 1:**
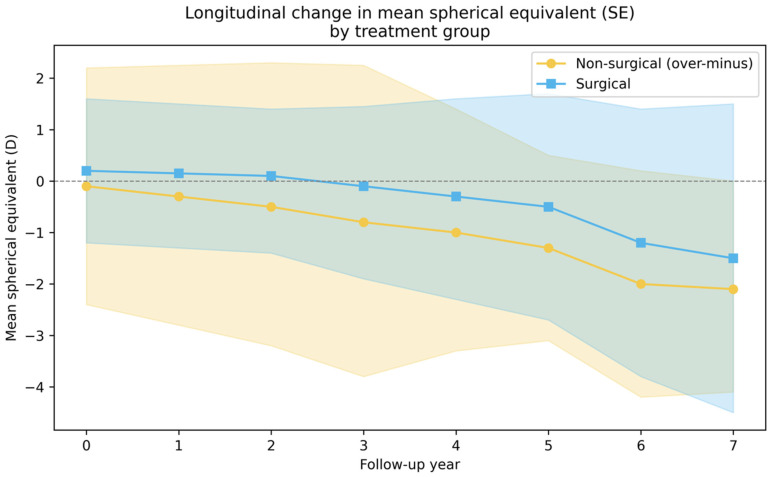
Longitudinal change in mean spherical equivalent (SE, diopters) in surgical and non-surgical (over-minus) groups during a 7-year follow-up.

Longitudinal analysis of spherical equivalent (SE) demonstrated a gradual myopic shift in both non-surgical (only OML) and surgically treated groups during the follow-up period. In the OML group, the baseline SE was −0.10 ± 2.34 D (median 0.00, IQR −0.75 to +1.00), declining to a mean of −2.10 ± 1.98 D (median −1.25) at year 7. This corresponded to an overall myopic shift of approximately 1.5–2.0 diopters (≈−0.20 to −0.30 D/year). Similarly, in the surgical group, baseline SE was +0.24 ± 1.44 D (median +0.44, IQR −0.50 to +1.00) and decreased to −1.51 ± 3.02 D (median −1.25) by year 7, reflecting a comparable myopic shift of ~1.5–2.0 D (≈−0.25 D/year) (Table 4). A linear mixed-effects model with repeated measures confirmed a significant main effect of time on SE (β = −0.25 D/year, *p* < 0.001), while the time × surgery interaction was not significant (β = +0.014 D/year, *p* = 0.541) (Table 4). This indicates that the rate of myopic progression did not differ between surgical and over-minus groups, and surgical intervention did not modify the natural trend toward myopia over time (Figure 1). All longitudinal analyses were conducted under a missing-at-random (MAR) assumption using mixed-effects modeling to accommodate unequal follow-up durations and incomplete refraction data.

Both groups showed a similar gradual myopic shift (β = −0.25 D/year, *p* < 0.001), with no significant difference in progression rate (*p* = 0.54). Shaded bands indicate ±½ SD.

### 3.5. Age-Related Control Analysis Among Surgically Treated Patients (n = 167)

Baseline control ability (ECS) was significantly related to both age at presentation and delay from onset to presentation (Table 5). For near control, mean age differed significantly across control groups (H = 10.285, *p* = 0.006), with older patients showing poorer control. The interval from deviation onset to hospital presentation was also clearly linked to control level: patients with poorer control presented after longer delays (H = 6.535 for near, *p* = 0.038; H = 8.580 for distance, *p* = 0.014).

### 3.6. Surgical Outcomes and Associated Factors

Among surgically treated patients (n = 167), the median preoperative deviation was 18 PD (range, 0–50) at near and 30 PD (range, 16–60) at distance. Postoperatively, alignment improved substantially at all follow-ups. On the first postoperative day, median deviations were 0 PD (IQR, 0) at near and 0 PD (IQR, 4) at distance, and these improvements remained stable through the 6-month and final follow-up visits (0 PD [IQR, 6] at near; 4 PD [IQR, 12] at distance).

At the final examination, surgical success—at both near and distant—was achieved in 65.3% (109/167) of surgically treated patients. Success rates did not differ significantly across IXT subtypes (*p* = 0.771). Neither age nor age at surgery affected outcomes. By procedure type, BLR showed a higher but nonsignificant success rate compared with R&R (81% vs. 61%, *p* = 0.108). Surgical dose (standard vs. augmented) also showed no significant association with success (*p* = 0.461) (Table 6).

### 3.7. Surgical Procedures and Postoperative Stability

The most commonly performed procedure was R&R, accounting for 70.1% of cases, followed by BLR in 25.7%. Single- and three-muscle surgeries were rarely indicated (<5%). The distribution of surgical procedures did not differ significantly across age groups (Fisher–Freeman–Halton = 5.321, *p* = 0.066), although a trend toward more frequent R&R procedures in older patients was observed. There were no gender-based differences in surgical type (χ^2^ = 0.038, *p* = 0.845). A significant association was found between IXT subtype and surgical approach (Fisher–Freeman–Halton = 11.652, *p* = 0.005). Post hoc evaluation indicated that this difference was primarily driven by a divergence in excess cases, which were more frequently treated with BLR (51.6%) than with R&R (48.4%). The mean age at surgery also differed significantly between groups: patients undergoing BLR were younger than those treated with R&R (6.5 ± 5.4 vs. 8.3 ± 5.6 years; *p* = 0.016) (Table 7).

During postoperative follow-up, ocular alignment and convergence remained stable in both surgical groups. Cochran’s Q test showed no significant change in alignment success over 6 months (BLR: *Q* = 0.900, *p* = 0.825; R/R: *Q* = 4.455, *p* = 0.216). In contrast, stereopsis improved significantly over time (BLR: χ^2^ = 8.566, *p* = 0.036; R/R: χ^2^ = 37.156, *p* < 0.001), with the greatest gains occurring between the early postoperative period (day 1–week 1) and later follow-up (month 1–month 6).

### 3.8. Reoperations

Among 167 surgically treated patients, 21 (12.6%) required a second operation for recurrent or residual deviation. The most common procedure at reoperation was R&R (38.1%), followed by oblique muscle surgery (33.3%) and single-muscle procedures (28.6%). Most second surgeries were performed with a standard surgical dose (72.2%), whereas augmented doses were used in 27.8% of cases. Of the reoperated patients, nine (42.9%) were male and 12 (57.1%) were female. Convergence was normal in 11 patients (52.4%) and impaired in 10 (47.6%). Preoperative ocular motility was free in the majority (71.4%), while abduction limitation grades 1 and 2 were observed in 9.5% and 19.1%, respectively. Oblique overaction was bilateral in 33.3%, unilateral in 23.8%, and absent in 42.9% of reoperated cases. Preoperative stereopsis was absent in 57.1% and present in 42.9% of patients undergoing a second surgery. Comparison of demographic characteristics between patients who had a single operation and those who underwent reoperation revealed no statistically significant differences (all *p* > 0.05). Rates of reoperation were similar between males (13.9%) and females (12.6%; *p* = 0.821). Delivery type (cesarean 9.8%, vaginal 14.8%, difficult delivery 100%) and gestational age (preterm 10.0%, term 14.5%) were not associated with surgical frequency (*p* = 0.077 and *p* = 1.000, respectively). Likewise, the presence of a family history of strabismus did not differ between groups (*p* = 0.081).

Notably, a single patient (0.6%) required three operations. This was a 10-year-old male with a history of mental–motor–retardation-related comorbidity, initially operated at 10 years of age and subsequently reoperated at 11 and 11.5 years.

### 3.9. Changes in Binocular Sensory Function During Follow-Up

At the time of initial clinical evaluation (N = 296), abnormal fusion was observed in 48 patients (16.2%), normal fusion in 233 patients (78.7%), and suppression in 15 patients (5.1%). At the final fusion assessment (N = 378), abnormal fusion was detected in nine patients (2.4%), normal fusion in 362 patients (95.8%), and suppression in seven patients (1.9%).

Most patients demonstrated stable or improved sensory fusion during follow-up. Notably, 91.3% of patients with initial abnormal fusion and 80% of those with initial suppression achieved normal fusion at final assessment, while fusion status remained stable in 99.1% of patients with normal baseline fusion. These findings suggest substantial improvement and overall preservation of binocular sensory function over time (Table 8).

A significant improvement in stereoacuity was observed over the follow-up period. The median stereopsis value improved from 200 arcsec (IQR: 2940) at the initial assessment to 80 arcsec (IQR: 40) at the final visit. This change was statistically significant according to the Wilcoxon signed-rank test (Z = −9.884, *p* < 0.001) (Table 9). And also, 50.9% of patients without baseline stereopsis acquired measurable stereopsis, whereas 97.7% of those with initial stereopsis maintained it (Table 10).

### 3.10. Kaplan–Meier Analysis of Surgical and Non-Surgical Outcomes

A Kaplan–Meier survival analysis was conducted for all 415 patients with IXT to evaluate the long-term probability of maintaining successful ocular alignment (defined as ≤±10 prism diopters at both near and distance). During follow-up, 245 patients (59.0%) experienced alignment failure (event), while 170 (41.0%) remained censored, reflecting sustained alignment or loss to follow-up. The median survival time (duration of successful alignment) was 70 ± 4.7 months (95% CI, 60.9–79.1). Cumulative success (survival) probabilities were 0.899 at 1 year, 0.563 at 5 years, and 0.302 at 10 years, indicating a gradual decline in stable alignment over time. Each downward step in the Kaplan–Meier curve represents a failure event, whereas tick marks denote censored observations (patients who remain successful at the last visit) (Table 8).

### 3.11. Univariate Log-Rank Analysis

Log-rank (Mantel–Cox) comparisons demonstrated that overall survival (success) differed significantly according to surgical status (χ^2^ = 98.781, *p* < 0.001) and baseline control level for both near and distance measures (ECS–near: χ^2^ = 4.847, *p* = 0.028; ECS–distance: χ^2^ = 11.324, *p* = 0.001). Patients who underwent surgery and those with better baseline control exhibited longer alignment survival times. No significant survival differences were observed with respect to sex (*p* = 0.171), age group (<7 vs. ≥7 years; *p* = 0.056), or prior OML therapy (*p* = 0.092), although younger age and absence of OML therapy showed borderline trends toward better outcomes (*p* < 0.25 threshold for inclusion in multivariate modeling).

### 3.12. Multivariate Cox Proportional Hazards Model

**Figure 2:**
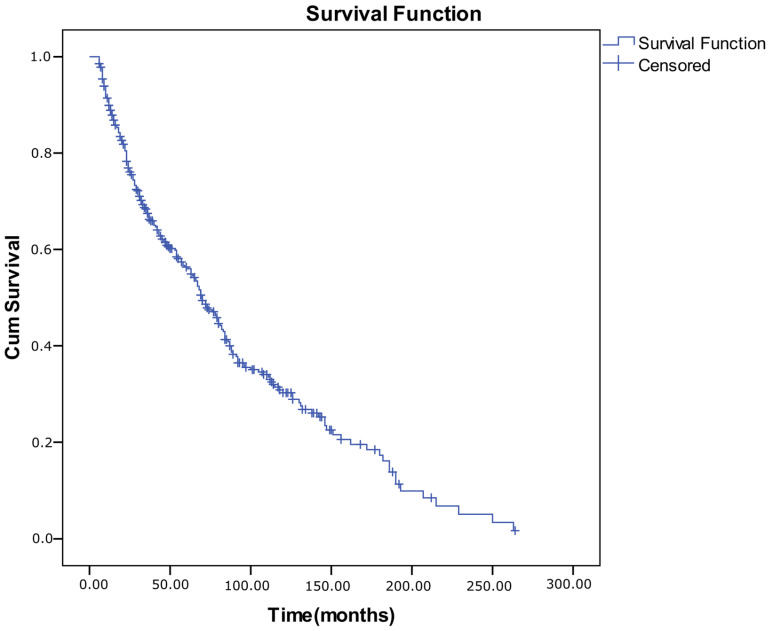
Kaplan–Meier survival curve for all intermittent exotropia patients (n: 415).

**Figure 3:**
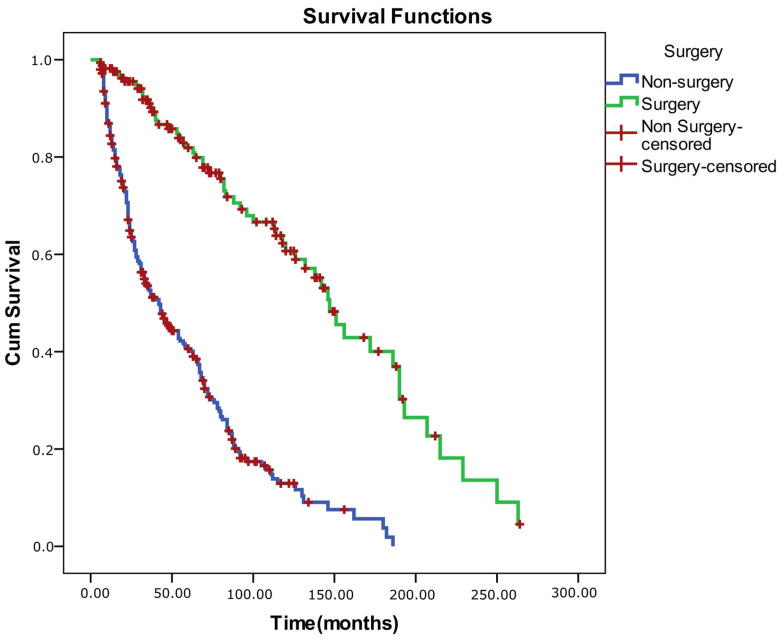
Kaplan–Meier survival curves comparing non-surgical and surgical management of intermittent exotropia.

**Figure 4:**
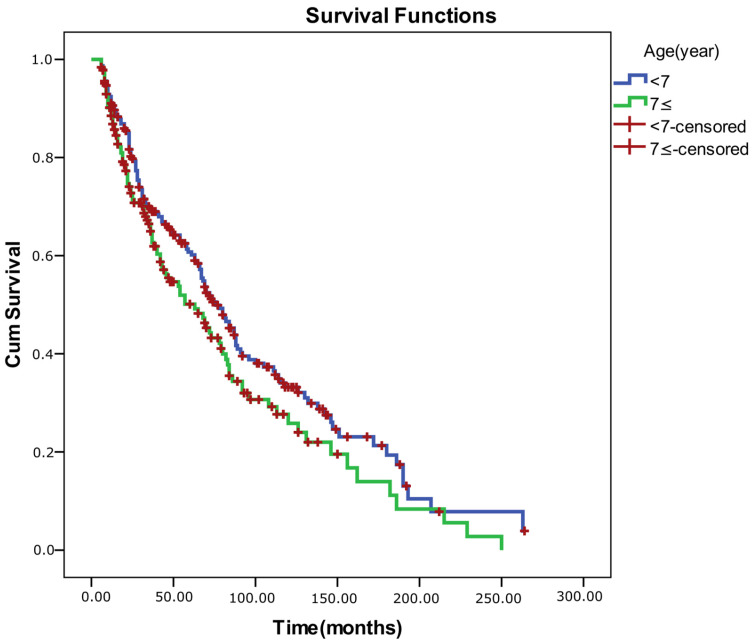
Kaplan–Meier survival curves by age at diagnosis in intermittent exotropia.

**Figure 5:**
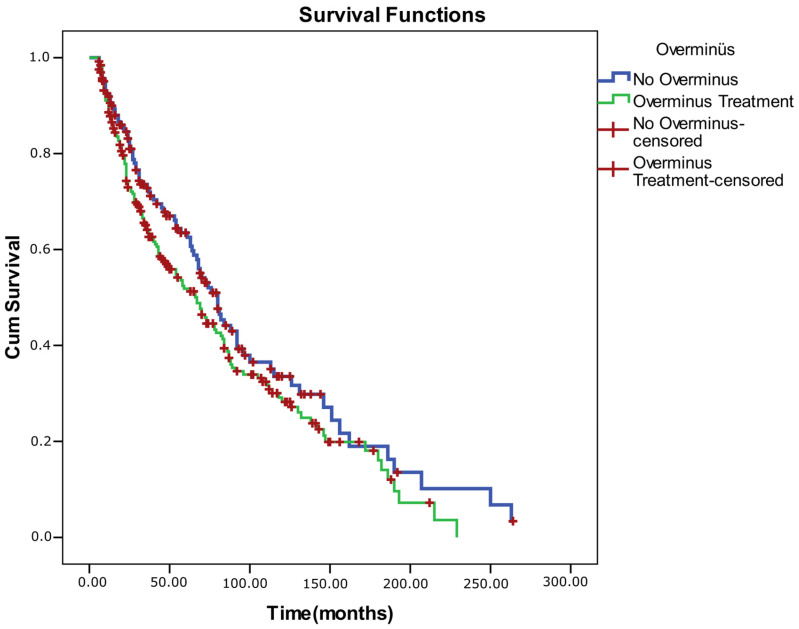
Kaplan–Meier survival curves comparing patients with and without over-minus lens therapy in intermittent exotropia.

Because the proportional hazards assumption was violated for sex (as confirmed by log–minus–log plots), a stratified Cox regression was performed by sex. The model showed good fit (−2 log likelihood = 2037.35; Omnibus test: χ^2^ = 113.19, *p* < 0.001). In the adjusted model, older age was associated with a significantly higher risk of alignment failure (HR = 1.040, 95% CI: 1.010–1.070, *p* = 0.008). Surgical intervention exerted a strong protective effect against recurrence (HR = 0.174, 95% CI: 0.120–0.252, *p* < 0.001). Conversely, poor distance control (ECS 3–5) increased the hazard of failure by 1.42-fold (HR = 1.421, 95% CI: 1.051–1.920, *p* = 0.022). Over-minus therapy and near control level were not independently associated with survival outcomes (*p* > 0.05) (Table 11). The Schoenfeld residual correlation test confirmed that all covariates satisfied the proportional hazards assumption (*p*-values > 0.05), indicating time-invariant hazard ratios. Survival graphs for the comparisons are also included below (Figure 2, Figure 3, Figure 4 and Figure 5).

## 4. Discussion

This large, real-world cohort study provides a detailed longitudinal assessment of IXT, combining comprehensive clinical profiling with advanced statistical modeling over an extended follow-up period. Our results illustrate the natural progression of IXT, the role of over-minus therapy, and key factors influencing surgical outcomes and alignment stability. Notably, 24.6% of non-surgically managed patients achieved spontaneous alignment, and 45.1% maintained acceptable control without requiring surgery. This finding supports the rationale for initial observation in selected cases with favorable control and stereopsis profiles, particularly in younger children. Over-minus therapy proved effective in delaying surgical intervention, without adversely affecting long-term alignment or accelerating myopic progression. Importantly, surgical success was not clearly linked to age at surgery or IXT subtype. However, survival analysis across the entire cohort showed that older age at presentation was associated with a significantly higher risk of alignment failure, whereas surgical treatment substantially reduced this risk. It appears that early diagnosis and timely intervention are critical to maintaining long-term ocular alignment.

The timing of treatment in IXT remains one of the most debated aspects of its management. Intervention is often considered when parents observe that the deviation is manifest for a significant portion of waking hours—typically exceeding 50%—as this threshold indicates an increased risk of visual suppression and potential loss of binocular single vision [11]. In addition, psychosocial concerns and the onset of stereopsis deterioration are well-recognized clinical indications for surgery [11]. However, no definitive consensus exists regarding the optimal timing for surgery. While some clinicians recommend delaying surgery until after age 4, others advocate for earlier intervention in cases with poor fusional control or large-angle deviations (typically >40–45 PD), irrespective of age [12]. Given that visual development is generally considered complete by age 7, procedures performed before this age may carry a greater risk of overcorrection, potentially leading to suppression, amblyopia, or loss of stereopsis [11]. However, some studies suggest that surgery performed between the ages of 3 and 5 can yield favorable motor outcomes [13]. In our cohort, age at surgery was not clearly linked to surgical success, and outcome comparisons across different age groups showed no statistically meaningful differences. This is consistent with a recent meta-analysis that found no universal benefit of early versus late surgery across all patients, although certain subgroups (e.g., children <4 years, BLR procedures) may derive some benefit [8]. Nonetheless, our survival analysis across the entire IXT cohort (including both surgical and non-surgical patients) revealed that older age at initial presentation was independently associated with an increased risk of alignment failure. This suggests that while age at the time of surgery may not be a critical determinant of surgical outcome, age at diagnosis and intervention—even when non-surgical—plays a vital role in long-term control.

Our study examined age-related differences in baseline control ability, as measured by the ECS, among surgical candidates. Analysis of the relationship between age and ECS scores at initial presentation revealed that near control was significantly poorer in older children compared to younger ones, whereas distance control showed no meaningful correlation with age. At first glance, this finding might suggest that near control deteriorates before distance control. However, the underlying mechanism likely reflects the natural course of IXT: distance control is typically the first to weaken in early childhood as fusional stability at distance fixation declines, so statistical differences with age are not readily apparent [9]. Accordingly, the poorer near control observed in older patients suggests that many have progressed beyond the early stage of distance instability and now exhibit both distance and near control loss—though the latter becomes more prominent with age. By adolescence, distance control is generally poor, and near control deterioration becomes the dominant feature. This shift from distance-dominant to near-dominant instability parallels the natural evolution of IXT and may explain why older patients more frequently present with convergence-insufficiency-like patterns and greater surgical need [14]. In our patients aged ≥15 years, convergence insufficiency further increased while pseudo-divergence excess decreased. Such a progression is consistent with reports that older children and adolescents with IXT often demonstrate near-dominant control deficits, characteristic of convergence-insufficiency patterns [15]. Convergence-insufficiency symptoms typically become more apparent during adolescence and adulthood due to increased visual demands such as reading, screen use, and prolonged near work. Older patients tend to report symptoms more reliably and cooperate better with convergence testing, likely contributing to higher CI detection rates, a trend also reported in large cohort studies such as Kim et al. [15]. Moreover, patients who presented later after symptom onset had significantly poorer ECS scores at their initial examination, suggesting that prolonged delays in ophthalmic evaluation allow fusional control to deteriorate further. This observation aligns with recent evidence showing that delayed presentation and older age are associated with greater control loss and poorer sensory outcomes [14]. Together, these results emphasize that control deterioration in IXT is primarily time-dependent rather than purely age-related [5].

IXT can be managed through surgical, non-surgical, or combined treatment approaches. Among conservative methods, OML therapy has emerged as the most effective option for improving control and reducing deviation, according to a recent meta-analysis [1].

In our cohort, OML therapy was the most commonly used non-surgical approach, with 252 patients trialed. Its therapeutic effect is primarily achieved through stimulating accommodation and accommodative convergence, thereby improving fusional control and reducing the angle of deviation [16,17]. Furthermore, OML therapy may counterbalance excessive accommodative effort exerted by patients attempting to prevent exotropic episodes, thus maintaining image clarity while enhancing ocular alignment [18]. Clinical outcomes from prior studies support the efficacy of OML therapy. Bayramlar et al. [19] reported that 84% of patients demonstrated improved control scores and a mean reduction in deviation from 25 PD to 18 PD over 18 months. Similarly, Abri Aghdam et al. [20] found that 66.8% of patients experienced significant improvement after one year of OML use. In younger children under seven, Alizadeh et al. [21] observed that 79.2% of patients responded successfully to OML treatment, with minimal progression of exotropia.

Concerns have been raised regarding whether prolonged OML use accelerates myopic progression [22]. However, a recent study reported no significant myopic shift among children receiving OML therapy, suggesting that this approach may be safe in terms of refractive development [21]. Similarly, in our cohort, patients treated with over-minus lenses showed a mean myopic shift of approximately −0.25 diopters per year, a rate comparable to physiological refractive changes in the general pediatric population. Importantly, our mixed-effects model revealed no significant time × surgery interaction (β = +0.014 D/year, *p* = 0.541), indicating that neither surgery nor OML therapy significantly altered the natural trajectory of myopic progression.

The degree of over-minus correction remains a matter of clinical debate. Most protocols advocate for a range of −1.00 to −3.00 D based on cycloplegic refraction [12,16]. The optimal power is determined by balancing visual clarity and maximal control improvement. Rowe et al. [23] proposed an incremental titration method, starting with −0.50 D lenses and increasing until control is achieved. In the PEDIG study, mean exotropia angle decreased from 20.6 ± 6.3 PD to 16.7, 14.0, and 11.4 PD with −1.00 D, −2.00 D, and −3.00 D lenses, respectively. Most children showed improvement at each level, with minimal cases of unchanged or worsened deviation [24]. The mean OML prescription in our clinic was −1.25 D, a milder correction compared to what was noted in the ranges of earlier studies [12,16,24]. This conservative dosing strategy may contribute to the absence of excessive myopic shift in our patients. In our clinical protocol, over-minus or under-correction is titrated gradually using dynamic retinoscopy to ensure that accommodative demand does not exceed the patient’s fusional capacity [25]. This careful approach helps minimize the risk of potential complications such as consecutive esotropia or asthenopia, which may arise if excessive accommodation is required to maintain alignment [21].

Our analysis revealed that patients receiving over-minus therapy typically underwent surgery at a younger chronological age yet experienced a longer interval between initial presentation and surgical intervention. This pattern likely reflects a selection bias, where OML therapy is preferentially prescribed to patients with larger baseline deviations and poorer control—characteristics associated with earlier surgical need. Nevertheless, the extended preoperative period suggests that OML may serve as an effective temporizing strategy, allowing clinicians to delay surgery without compromising long-term alignment outcomes [16,23].

The likelihood of receiving over-minus therapy also varied significantly by IXT subtype (*p* = 0.002), with the highest usage observed in pseudo-divergence excess cases (71.9%) and the lowest in the basic type (51.7%). This distribution aligns with clinical expectations, as patients with pseudo-divergence excess typically demonstrate better control at distance but reduced control at near, likely due to insufficient fusional convergence. As such, they may respond more favorably to accommodative-based interventions such as OML therapy [17].

Some studies suggest that significant hypermetropia or low-to-moderate myopia can contribute to IXT by reducing accommodative convergence, thereby permitting outward deviation [17]. Anisometropia likewise may disrupt sensory fusion, which in turn interferes with the sensorimotor fusion relationship and ultimately promotes strabismus. In this context, correcting such refractive errors may augment sensory fusion, indirectly enhance motor fusion, and stabilize ocular alignment control [17]. Han et al. [26] found that uncorrected refractive error detrimentally affects stereoacuity in children with IXT, and that impaired stereopsis is associated with poorer long-term IXT control. In myopic patients, full refractive correction is often recommended because it reduces the demand for accommodative convergence. In hypermetropic children, although some clinicians argue that hyperopia may increase both the frequency of deviation and the magnitude of misalignment, many cases with high hypermetropia demonstrate improvement after optical correction [17]. A mechanistic explanation posits that, in high hypermetropia, reduced accommodative reserve may lead to insufficient convergence stimulation, thereby triggering exotropia [25]. In such cases, the resultant blur from inadequate accommodation further destabilizes retinal image clarity. It promotes manifest strabismus—hence, optical correction may improve retinal image quality and thereby enhance control of alignment [27]. In our series, 61 patients (14.7%) demonstrated spontaneous improvement of IXT solely with correction of the existing refractive error. Furthermore, evidence from the PEDIG indicates that children with IXT aged 12–35 months may be safely observed without immediate intervention, as no significant progression was seen. Thus, when a patient is able to control the deviation, shows no worsening of angle over successive visits, and lacks functional or cosmetic issues, a strategy of closely monitored observation may be appropriate [24].

In our cohort, no patient without amblyopia received occlusion therapy, as concerns have been raised that patching may disrupt binocular fusion mechanisms. Occlusion may inherently eliminate simultaneous binocular input, thereby limiting the opportunity for normal sensory fusion. Suwal et al. [28] recently demonstrated that, even in amblyopic children, those who received monocular occlusion therapy exhibited significantly lower stereopsis levels than those treated with binocular-based approaches such as active vision therapy. This finding suggests that the interruption of binocular visual experience—such as with occlusion—may have a detrimental impact on sensory fusion. Cotter et al. [29] further compared part-time patching (3 h/day) with observation in previously untreated children aged 3–10 years and found no significant differences in control or motor outcomes after six months of follow-up. According to the PEDIG, deterioration of IXT was rare over a six-month follow-up period in children aged 12 to 35 months, regardless of whether they received part-time occlusion therapy or were simply observed [30].

In our surgical cohort of 167 patients (40.2% of the total population), the single-surgery success rate at final follow-up was 64.4%, with a median follow-up of 40 months. Across the cohort, the overall reoperation rate was 13.2%, consistent with the literature [7]. Our extended follow-up provides a more comprehensive view of long-term outcomes than previous studies. For instance, Lino et al. [1] reported a higher success rate of 92.2%, though their follow-up was limited to 6 months and included only patients who underwent BLR surgery. Other studies have reported more modest short-term success rates—for example, Chew et al. [31] noted a 75% success rate at 1 week postoperatively, while Lee et al. [32] noted a 61.4% success rate at 1 month in a separate cohort.

In our prespecified sub-analysis excluding patients who underwent combined oblique surgery, we observed a higher rate of near-alignment success in the R&R group compared to the BLR group. This difference is likely due to the medial rectus resection component of the R&R procedure, which enhances convergence and is especially advantageous for patients with near control deficits or convergence-insufficiency-type exotropia. In contrast, BLR primarily reduces divergence and is generally more effective for controlling distance deviations, especially in cases with divergence excess or pseudo-divergence excess patterns [33,34]. In our cohort, 70.1% of patients underwent R&R, while 25.7% received BLR. A statistically significant relationship was observed between IXT subtype and surgical technique, with divergence-type deviations more often managed using BLR. This approach is consistent with literature reports showing up to 86% success with BLR, especially in divergence excess patterns [35]. Importantly, despite differences in near-alignment outcomes, overall surgical success—considering both near and distance alignment—did not differ significantly between the R&R and BLR groups in our study. A recent meta-analysis similarly found comparable efficacy and risk profiles for both techniques in basic-type IXT [34].

While motor alignment remained stable between postoperative day 1 and month 6, stereopsis significantly improved over this period, suggesting that sensory outcomes may continue to benefit from surgical realignment even as motor outcomes begin to plateau. This aligns with findings by Srimanan and Keokajee [36] who reported that while favorable ocular alignment was typically maintained during the first six months postoperatively, improvements in binocular function—particularly stereopsis—were observed to continue beyond this initial phase. In our cohort, subgroup analysis revealed that both BLR and R&R procedures yielded significant gains in stereopsis, suggesting that surgical realignment, regardless of technique, supports sensory rehabilitation when motor success is achieved [37].

Kushner was among the first to propose that augmenting BLR dosages may improve surgical outcomes in basic-type IXT without significantly increasing the risk of overcorrection [38]. Subsequent studies have supported this approach, showing that increasing the lateral rectus recession by 1.0–2.5 mm or adjusting the target deviation by an additional 5 PD can enhance long-term motor success without a significant increase in the rate of consecutive esotropia [39,40]. Kim et al. [39] emphasized that augmented BLR surgery may be especially beneficial in divergence excess-type IXT. Similarly, medial rectus resection augmentation in R&R procedures—typically by 1 mm—has also been associated with improved long-term outcomes, again without a significant increase in the risk of overcorrection [41]. In our study, augmented dosages were applied in 62 patients, encompassing both BLR and R&R procedures. Success rates were high in both groups—75% in the augmented BLR group and 66.7% in the augmented R&R group—though the difference did not reach statistical significance. Interestingly, in a recent study by Lino et al. [1] Although direct comparison between augmented and standard dosages did not yield statistically significant differences, logistic regression analysis suggested a possible association between augmented dosing and decreased surgical success. These mixed findings highlight the need for careful patient selection and individualized dosing strategies, especially when planning enhanced surgical interventions in IXT.

In our study, survival analysis confirmed this pattern, showing a steady decrease in alignment stability during long-term follow-up. Specifically, the proportion of patients maintaining orthophoria declined progressively, consistent with prior findings reported by Kopmann et al. [5] who demonstrated that deviation angles tend to recur and shift outward over time following initial surgical success. Further statistical modeling revealed that surgical intervention was strongly protective against recurrence. Conversely, older age at baseline was associated with a higher risk of alignment failure, as was poor baseline distance control, which increased the risk of failure by 1.42 times. These findings emphasize the importance of early diagnosis, timely surgical intervention, and careful preoperative evaluation of control scores, particularly for distance deviation [31,42].

This study has several limitations that warrant consideration. First, although the cohort size is large and the follow-up period is extended, its retrospective design carries inherent risks of selection bias and incomplete data documentation. Second, clinical decisions regarding both non-surgical and surgical interventions—such as the choice and dosage of over-minus lens therapy, timing of surgery, and surgical approach—were guided by real-world discretion rather than a uniform protocol. This enhances the generalizability of our findings but limits direct comparability across treatment arms. Third, while stereoacuity outcomes were analyzed, detailed binocular function assessments (e.g., fusional vergence amplitudes and distance-specific control scores) were not consistently available. Although standardized protocols were employed, the involvement of multiple examiners and surgeons may have introduced some variability. This is acknowledged as a potential source of bias. Lastly, psychosocial and patient-reported outcomes were not assessed, despite their relevance to understanding treatment impact. Despite these limitations, the study offers meaningful real-world insights into the clinical course and management of intermittent exotropia. The findings may help inform individualized treatment decisions, particularly in balancing timely surgical intervention with observation in selected cases.

## 5. Conclusions

This large, real-world cohort study offers comprehensive insights into the longitudinal course and management of IXT. Our findings confirm that a substantial proportion of children can be safely observed or managed non-surgically, with some even achieving spontaneous alignment. Early clinical presentation was associated with delayed surgical intervention, likely reflecting both timely detection and a longer window for conservative management. Furthermore, even low-dose OML therapy—averaging only −1.25 D in our cohort—proved effective in delaying surgery, particularly in patients with pseudo-divergence excess, without accelerating myopic progression. Surgical alignment outcomes were not dependent on chronological age, and although motor alignment stabilized early postoperatively, stereoacuity continued to improve, underscoring the value of long-term follow-up in capturing sensory recovery. These findings reinforce the need for individualized, phenotype-driven treatment strategies and support a conservative approach in selected patients. Future prospective studies are warranted to further refine treatment indications and optimize both motor and sensory outcomes in IXT.

## Figures and Tables

**Table 1:** Baseline demographic and clinical characteristics of patients with intermittent exotropia (n = 415).

Continuous Variables		
Variable	Mean ± SD	Median (IQR)
Age at onset (years)	3.7 ± 2.9	3 (3.5)
Age at presentation (years)	6.8 ± 4.9	6 (5.0)
Follow-up duration (months)	53.2 ± 47.8	40 (63)
Categorical variables		
Variable	Category	n (%)
Sex	Female	243 (58.6)
	Male	172 (41.4)
Gestational age	Term	317 (76.4)
	Preterm	98 (23.6)
Birth type	Vaginal	213 (51.3)
	Cesarean	127 (30.6)
	Unknown *	75 (18.1)
Family history of strabismus/amblyopia	Positive	74 (17.8)
	Negative	341 (82.2)
IXT subtype	Basic	201 (48.4)
	Pseudo-divergence excess	139 (33.5)
	Divergence excess	59 (14.2)
	Convergence insufficiency	16 (3.9)

* Missing perinatal data due to incomplete records.

**Table 2:** Distribution of IXT subtypes by age groups.

Age Group Years	XT Types—Simple	XT Types—Divergence	XT Types—Convergence	XT Types—Pseudo-D	Test Value	*p*
≤4	59 (43.4%)	25 (18.4%)	1 (0.7%)	51 (37.5%)	24.735	0.002
4–6 years	41 (44.6%)	13 (14.1%)	1 (1.1%)	37 (40.2%)		
7–14 years	84 (52.8%)	18 (11.3%)	10 (6.3%)	47 (29.6%)		
≥15 years	17 (60.7%)	3 (10.7%)	4 (14.3%)	4 (14.3%)		

Statistical test: Fisher–Freeman–Halton Exact, *p* < 0.001.

**Table 3:** Effect of over-minus therapy on surgical timing (n = 167 surgically treated patients).

Parameter	Group	n	Mean ± SD	Median (IQR)	Statistical Test	*p*-Value
Age at surgery (years)	No OML	84	11.2 ± 7.1	10 (7–14)	−3.329	0.001
	OML	83	7.9 ± 3.5	7 (5–9)		
Time from first visit to surgery (years)	No OML	84	1.34 ± 2.07	0.75 (0–2)	−2.075	0.038
	OML	83	1.83 ± 2.40	1 (0–2)		

OML = over-minus lens therapy. Data are presented as mean ± standard deviation and median (interquartile range). Mann–Whitney U test was used for group comparisons.

**Table 4:** Summary of longitudinal spherical equivalent (SE) changes in surgical and non-surgical (over-minus) groups over 7 years of follow-up.

Follow-Up Year	Over-Minus Group (Mean ± SD)	Surgical Group (Mean ± SD)	Mean Difference (95% CI)
Baseline	−0.10 ± 2.34	+0.24 ± 1.44	0.34 (−0.21 to 0.89)
Year 3	−1.05 ± 2.03	−0.84 ± 2.41	0.21 (−0.48 to 0.89)
Year 5	−1.61 ± 2.06	−1.38 ± 2.63	0.23 (−0.61 to 1.08)
Year 7	−2.10 ± 1.98	−1.51 ± 3.02	0.59 (−0.42 to 1.60)
β (slope)	−0.25 D/year, *p* < 0.001	−0.26 D/year, *p* < 0.001	Interaction *p* = 0.541

Linear mixed-effects model with random intercept and slope per subject. Missing data handled under MAR assumption. β denotes mean annual change in SE (D/year). *p*-values derived from Wald tests. No significant time × surgery interaction observed (*p* = 0.541).

**Table 5:** Relationship between baseline control (ECS), age at presentation, and delay from onset to presentation.

ECS Group		n	Age at Presentation (Years)			Delay from Onset to Presentation (Years)		
			Mean ± SD	Median (IQR)	*p*	Mean ± SD	Median (IQR)	*p*
ECS–Near	Good (1–2)	93	6.83 ± 4.59	6 (4–9)	0.006 * (H = 10.285)	3.41 ± 4.02	2 (1–5)	0.038 * (H = 6.535)
	Moderate (3)	54	8.49 ± 5.82	7 (5–11)		4.79 ± 5.13	3 (1–6)	
	Poor (4–5)	19	11.92 ± 7.91	10 (7–16)		6.02 ± 5.10	5 (3–9)	
ECS–Distance	Good (1–2)	18	6.80 ± 6.10	6 (4–9)	0.155 (H = 3.731)	3.52 ± 5.50	2 (1–5)	0.014 * (H = 8.580)
	Moderate (3)	73	7.42 ± 4.75	7 (5–10)		3.38 ± 4.15	2 (1–5)	
	Poor (4–5)	74	8.83 ± 6.30	8 (6–12)		5.13 ± 4.70	4 (2–8)	

Data are presented as mean ± standard deviation and median (interquartile range, q25–q75). Analyses were restricted to surgically treated patients with complete data (n = 160–167). Kruskal–Wallis H tests were used for group comparisons. * *p* < 0.05 indicates statistical significance. Post hoc (Dunn–Bonferroni) analyses showed that patients with poor control were significantly older and presented later than those with good control.

**Table 6:** Surgical success and associated factors among surgically treated patients (n = 167).

Parameter	Metric	Successful (n = 109)	Unsuccessful (n = 58)	*p*-Value
Overall success rate	n (%)	109 (65.3%)	58 (34.7%)	—
Age (years) *	Mean ± SD/Median (IQR)	7.72 ± 5.42/6 (6)	8.28 ± 6.13/6.25 (7)	0.737
Surgical age (years) *	Mean ± SD/Median (IQR)	9.30 ± 5.65/8 (5)	9.89 ± 6.06/9 (6.25)	0.757
IXT subtype †	n (%)			0.771
Basic		49 (66.2%)	25 (33.8%)	
Divergence excess		21 (67.7%)	10 (32.3%)	
Pseudo-divergence excess		32 (59.3%)	22 (40.7%)	
Convergence insufficiency		1 (100%)	0 (0%)	
Surgical type ‡	n (%)			0.108
BLR		32 (80.7%)	11 (19.3%)	
R/R		71 (60.7%)	46 (39.3%)	
Surgical dose ‡	n (%)			0.461
Standard		43 (66.1%)	19 (33.9%)	
Augmented		65 (63.7%)	37 (36.3%)	

* Mann–Whitney U test. † Fisher–Freeman–Halton Exact test. ‡ Pearson Chi-square test. Data are presented as mean ± standard deviation, median (interquartile range), or number (percentage) as appropriate. Surgical success was defined as residual deviation ≤ ±10 prism diopters (PD) at both near and distance at the final follow-up. BLR: bilateral lateral rectus recession; R/R: unilateral recession–resection.

**Table 7:** Distribution and clinical characteristics of surgical procedures in intermittent exotropia (n = 167).

Parameter	Category	BLR n (%)	R/R n (%)	Statistical Test	*p*-Value
Age group (years) *	≤6 (n = 84)	29 (34.5%)	55 (65.5%)	5.321	0.066
	7–14 (n = 60)	12 (20.0%)	48 (80.0%)		
	≥15 (n = 16)	2 (12.5%)	14 (87.5%)		
Gender †	Male (n = 69)	18 (26.1%)	51 (73.9%)	0.038	0.845
	Female (n = 91)	25 (27.5%)	66 (72.5%)		
IXT subtype *	Basic (n = 74)	14 (18.9%)	60 (81.1%)	11.652	0.005
	Divergence excess (n = 31)	16 (51.6%)	15 (48.4%)		
	Pseudo-divergence excess (n = 54)	13 (24.1%)	41 (75.9%)		
	Convergence insufficiency (n = 1)	0 (0%)	1 (100%)		
Mean surgical age (years) ‡	Mean ± SD/Median (IQR)	6.5 ± 5.4/5 (6)	8.3 ± 5.6/7 (6)	−2.401	0.016

* Fisher–Freeman–Halton exact test. † Pearson Chi-square test. ‡ Mann–Whitney U test. Data are presented as number (percentage), mean ± standard deviation, or median (interquartile range) as appropriate. BLR = bilateral lateral rectus recession; R/R = unilateral recession–resection.

**Table 8:** Transition of fusion status between initial and final evaluations.

		Final Fusion			Total
		Abnormal Fusion	Fusion	Suppression	
Initial Fusion	Abnormal Fusion	3 (6.5%)	42 (91.3%)	1 (2.2%)	46
	Fusion	1 (0.4%)	229 (99.1%)	1 (0.4%)	231
	Suppression	1 (6.7%)	12 (80%)	2 (13.3%)	15
		5 (1.7%)	283 (96.9%)	4(1.4%)	292

**Table 9:** Changes in stereoacuity from initial to final evaluation.

	N	Mean	Standard Deviation	Min	Max	Median (IQR)	Test Value	*p*
Initial Stereopsis	255	1.015.69	1.314.20	40	3000	200 (2940)	−9.884	<0.001
Final Stereopsis	255	244.71	622.48	40	3000	80 (40)		

Note: Test value: Wilcoxon test; *p* < 0.05 is considered statistically significant.

**Table 10:** Comparison of initial and final stereopsis status.

		Final Stereopsis			*p*
		Absent	Present	Total	
Initial Stereopsis	Absent	26 (49.1%)	27 (50.9%)	53 (16.9%)	0.000
	Present	6 (2.3%)	255 (97.7%)	261 (83.1%)	
	Total	32 (10.2%)	282 (89.8%)	314 (100%)	

Note: Test value: McNemar test; *p* < 0.05 is considered statistically significant.

**Table 11:** Kaplan–Meier and Cox regression analysis of alignment survival in intermittent exotropia (n = 415).

Variable	Category/Metric	n	Events	Censored (%)	Median Survival (Months, 95% CI)	χ^2^ (Log-Rank)	*p*-Value	Hazard Ratio (HR, 95% CI)	*p* (Cox)
Overall	—	415	245	41.0	70 (60.9–79.1)	—	—	—	—
Sex	Male vs. Female	172/243	92/153	46.5/37.0	78 (57.3–98.7)/70 (60.6–79.4)	1.874	0.171	—	—
Surgery	Yes vs. No	167/248	58/187	65.3/24.6	147 (129.3–164.7)/42 (31.8–52.2)	98.781	<0.001	0.174 (0.120–0.252)	<0.001
ECS–Near	Good (1–2) vs. Poor (3–5)	307/105	192/51	37.5/51.4	69 (55.7–82.3)/79 (58.5–99.6)	4.847	0.028	1.147 (0.806–1.633)	0.446
ECS–Distance	Good (1–2) vs. Poor (3–5)	182/229	125/118	31.3/48.5	58 (41.7–74.3)/84 (70.6–97.4)	11.324	0.001	1.421 (1.051–1.920)	0.022
Age group	<7 vs. ≥7 years	228/187	138/107	39.5/42.8	76 (63.6–88.4)/63 (43.6–82.4)	3.649	0.056	1.040 (1.010–1.070)	0.008
Over-minus therapy	Yes vs. No	252/163	156/89	37.1/45.4	67 (53.0–81.0)/80 (67.5–92.6)	2.843	0.092	1.089 (0.824–1.438)	0.550

Data are presented as median survival time with 95% confidence intervals derived from Kaplan–Meier estimates. Hazard ratios (HR) and *p*-values correspond to the stratified Cox regression model (stratified by sex). Log-rank test: Mantel–Cox method. Significant results at *p* < 0.05.

## Data Availability

The original contributions presented in this study are included in the article. Further inquiries can be directed to the corresponding author.

## Abbreviations

The following abbreviations are used in this manuscript:

BLR	Bilateral Lateral Rectus Recession
CI	Confidence Interval
ECS	Eye Control Scale
HR	Hazard Ratio
IQR	Interquartile Range
IXT	Intermittent Exotropia
LMM	Linear Mixed-Effects Model
MAR	Missing At Random
OML	Over-Minus Lens (Over-minus Lens)
PD	Prism Diopters
PEDIG	Pediatric Eye Disease Investigator Group
R&R	Recession–Resection
SD	Standard Deviation
SE	Spherical Equivalent
SPSS	Statistical Package For The Social Sciences (IBM SPSS Statistics)
